# The effects of background music tempo on consumer variety-seeking behavior: the mediating role of arousal

**DOI:** 10.3389/fpsyg.2023.1236006

**Published:** 2023-08-29

**Authors:** Wenwen Sun, En-Chung Chang, Yifan Xu

**Affiliations:** School of Business, Renmin University of China, Beijing, China

**Keywords:** background music, music tempo, variety-seeking, arousal, music familiarity

## Abstract

Diversified purchases of consumers can help companies balance sales and inventories, which is of great significance to company profits. While existing research has explored the internal factors and external factors that influence consumers’ variety-seeking behavior, little is known about the impact of background music, an important environmental cue in retail establishments, on consumer variety-seeking behavior. The present research investigates the influence of background music tempo on consumer variety-seeking behavior, along with its underlying mechanism and boundary condition. Five experiments revealed that background music tempo affects consumers’ variety-seeking behavior (Study 1a, 1b & 4). Specifically, fast-tempo background music increases consumers’ variety-seeking behavior (Study 1b). Arousal mediates the main effect (Study 2), as fast-tempo background music increases consumers’ variety-seeking behavior by enhancing consumers’ arousal. Moreover, participants’ familiarity with the background music moderates the impact of background music tempo on consumer variety-seeking behavior (Study 3). Only when consumers have a high degree of familiarity with the background music they listen to, the tempo of the background music will have a significant impact on their variety-seeking behavior. These findings provide important theoretical contributions and management implications.

## 1. Introduction

The intensifying market competition has driven companies to continuously expand the length and width of product lines, increase product diversity to cover a wider range of consumer market segments, and enhance market share. New brands and products are emerging rapidly, making consumers overwhelmed. For instance, Pocky (A cookie brand from Glico) has launched more than 100 different flavors around the world. Likewise, the well-known fast-fashion brand Zara maintains a staggering rate of updating approximately 12,000 new items each year, with new arrivals appearing twice a week. However, the rapid increase in product categories has also brought pressure on inventory management. How to increase the diversification of consumers’ purchases and encourage customers to switch between brands and products more frequently to balance the sales of each product category has become an important concern for marketers.

Variety-seeking behavior refers to the tendency of consumers to seek variety in their product and service choices (Ratner and Kahn, 2002). A great deal of research has been conducted around variety-seeking behavior, delving into the internal and external factors that influence consumers’ variety-seeking behavior (see Zhang (2022) for a literature review). However, while existing research has explored some of the environmental factors that influence consumers’ variety-seeking (Levav and Zhu, 2009; Mohan et al., 2012; Deng et al., 2016), little is known about the impact of background music on consumers’ variety-seeking behavior.

Background music is an important environmental cue which affects consumers’ emotions, attitudes, and purchasing behaviors (Alpert and Alpert, 1990; Andersson et al., 2012; Michel et al., 2017). Whether we step into a shopping mall or a restaurant, we are likely to hear the background music played by the merchants. According to previous studies, the mood and meaning conveyed by music is determined by musical structural features such as pitch, timbre, and tempo (Scherer et al., 2001). Among them, music tempo is the most important structural feature that influences the expression of music (Juslin and Lindström, 2010). Can background music tempo affect consumer variety-seeking behavior? To answer this question, we explore the effect of background music tempo on consumers’ variety-seeking behavior and examine the mediating role of arousal level and the moderating role of musical familiarity in this effect through a series of experiments.

The current research has significant theoretical value. We not only identify background music tempo as a new environmental factor influencing consumers’ variety-seeking behavior, but also contribute a new outcome variable of music tempo. In addition, the findings of this research provide marketers with guidance on how to influence consumers’ diverse purchases by selecting and using background music, which has important practical implications.

## 2. Literature review and hypotheses

### 2.1. Variety-seeking behavior

Variety-seeking behavior refers to the tendency of consumers to seek variety in their product and service choices (Mohan et al., 2012). Variety-seeking behavior is a form of switching behavior characterized by the frequency of transitions among different products, brands, consumption activities, and services in a sequential decision situation or the number of different products or services chosen from a fixed choice set in a single choice situation (Ratner and Kahn, 2002; Sevilla et al., 2019).

Variety-seeking behavior is a tool for consumers to satisfy their needs for stimulation (Etkin and Mogilner, 2016; Sevilla et al., 2016). Variety means change. Consumers feel stimulated and excited when they choose something different. Menon and Kahn (1995) found that when individuals perceive their current level of stimulation to be lower than their optimal stimulation level, they increase their variety-seeking behavior. A study by Huang et al. (2019) revealed that sleepy consumers will engage in variety-seeking behaviors to enhance their arousal level to keep themselves awake.

Numerous researchers have explored the factors that influence consumer diversity seeking (Zhang, 2022). These factors mainly focus on individual and social factors, such as gender (Faraji-Rad et al., 2013), power state (Jiang et al., 2014; Wang and Jin, 2022), emotion states (Roehm and Roehm, 2005; Chuang et al., 2008), physiological states (Huang et al., 2019) and social environment (Ariely and Levav, 2000; Ratner and Kahn, 2002; Etkin, 2016). Only a few studies have explored the physical environment factors that affect variety-seeking. Deng et al. (2016) pointed out that the display format of products affects consumers’ variety-seeking behavior, with a horizontal display of merchandize increasing consumers’ variety-seeking tendency compared to a vertical display. Levav and Zhu (2009) found that consumers confined by space make more various choices to resist the invasion of their private space and seek freedom. Mohan et al. (2012) revealed that the store environment affects variety-seeking positively.

Although existing research has explored the impact of some environmental factors on consumers’ variety-seeking behavior, there has been a lack of research solely focusing on the influence of background music, an important physical environmental cue, on consumers’ variety-seeking behavior. In particular, the effect of different structural features of music on consumers’ variety-seeking behavior, such as music tempo.

### 2.2. Music tempo, arousal level, and consumer variety-seeking behavior

Music is an essential element in modern marketing practices. Extensive research has explored how background music in marketing environments affects consumers’ emotions, attitudes, and behaviors (Alpert and Alpert, 1990; Bruner, 1990; Areni, 2003; Andersson et al., 2012; Michel et al., 2017). Music consists of structural features such as rhythm, melody, timbre, and tempo, which significantly influence the emotional and semantic conveyance of music (Scherer et al., 2001). Juslin and Lindström (2010) found that music tempo is more powerful than other musical features in determining people’s response to music. Furthermore, music tempo is easier to identify, measure and modify than other structural features. Therefore, in the current research, we focus exclusively on the influence of music tempo on consumers’ behavior.

Tempo is the speed at which a piece of music is played and is usually measured in Beats Per Minute (BPM) (Bretherton et al., 2019). Generally, a tempo range of 70 to 110 BPM is the preferred tempo range (Holbrook and Anand, 1990). Dubé et al. (1995) categorized music with a tempo of 40–76 BMP as slow-tempo music, 77–107 BPM as moderate-tempo music, and 108–208 BPM as fast-tempo music.

Music tempo has a wide-ranging impact on individuals’ emotions, cognitions, and behaviors (Oakes, 2003; Xiao et al., 2020). For example, several studies have demonstrated that the tempo of background music influences consumers’ pace of action. Listening to fast-tempo music speeds up the rate at which people eat (Roballey et al., 1985), walk (Franěk et al., 2014), drive (Brodsky, 2002), and read (Su et al., 2023).

The music tempo is a determining structural feature in the influence of music on consumer arousal level (Gomez and Danuser, 2007; Liu et al., 2018). Arousal level is the degree to which a person feels excited, stimulated, alert, and active, and is an emotional dimension ranging from sleepiness to extreme excitement (Das and Varshneya, 2017). Arousal, similar to valence, is a significant component of emotion and is typically measured through self-report or physiological indicators (Russell, 1980; Russell et al., 1989). Fast-tempo music increases people’s arousal (Droit-Volet et al., 2013). Because fast-tempo music can convey more information and possess more stimulating elements that require more cognitive resources to process than slow-tempo music over the same period (Hahn and Hwang, 1999; Husain et al., 2002). Several studies have shown that fast-tempo music is associated with happiness or excitement, while slow-tempo music makes people feel calm, depressed, and sad (Webster and Weir, 2005; Hunter et al., 2010). The impact of music tempo on consumer emotional arousal is also reflected in some physiological indicators, such as breathing rhythm (Gomez and Danuser, 2007), heart rate (Mikutta et al., 2013), and skin conductivity level (Dillman Carpentier and Potter, 2007). In summary, the music tempo has an impact on people’s emotional arousal, whereby fast-tempo music increases people’s arousal level compared to slow-tempo music.

The effect of music tempo on the level of consumer arousal will further influence their behavior. Previous research has shown that consumers’ need for stimulation changes with arousal levels. When consumers are in a high arousal state, their need for external stimuli becomes higher due to higher intrinsic stimulation levels (Di Muro and Murray, 2012; Gullo et al., 2019). Then consumers will employ stimulation-seeking behaviors to fulfill their heightened need for stimulation, such as exploratory behavior and approach behavior. For example, research by Ridgway et al. (1990) found that consumers with higher levels of arousal explore the retail environment more and interact more with others in the shopping context. Dubé et al. (1995) found that when consumer arousal is increased, consumers increase their interactions with sellers. Menon and Kahn’s (2002) study revealed that consumers who had high levels of arousal from their previous online shopping experience were more likely to engage in high-arousal activities in subsequent purchases, such as exploring shopping venues, learning about new products and stores, and responding more actively to promotions. As an effective way to compensate for consumer stimulation levels, variety-seeking behavior also can fulfill the stimulation needs of high-arousal consumers (Kahn, 1995; Etkin and Mogilner, 2016). Therefore, we posit that when consumers’ arousal level is increased by fast-paced background music, consumers will increase their variety-seeking behavior. The following hypotheses are proposed:

*H1*: The tempo of background music affects consumers’ variety-seeking behavior. Compared to slow-tempo background music, fast-tempo background music increases consumers’ variety-seeking behavior.

*H2*: Arousal level mediates the effect of background music tempo on consumers’ variety-seeking behavior. Fast-tempo background music enhances consumers’ arousal levels, which in turn increases consumers’ variety-seeking behavior.

### 2.3. The moderation of background music familiarity

Consumers’ familiarity with a piece of music depends on how often consumers are exposed to it or how much they know about it (Hee Park et al., 2014). The level of music familiarity influences how consumers perceive and process the music they hear (Hahn and Hwang, 1999; Scherer et al., 2001). People prefer music they are familiar with to music they are not familiar with (Zissman and Neimark, 1990; Schellenberg et al., 2008). Ali and Peynircioǧlu (2010) demonstrated that listeners’ familiarity with music increases the intensity of their emotional responses to the music. Pereira et al. (2011) employed neuroimaging techniques and observed that when individuals listened to familiar music compared to unfamiliar music, their brain regions associated with emotions (limbic, paralimbic, and reward circuitry) were more active. This finding suggests that the familiarity of music enhances listeners’ music fruition, leading listeners to focus their attention more on the timbre of instruments, variations in music harmony and rhythm, and becoming more easily moved by the melody.

Accordingly, we infer that the effect of music tempo on consumers’ arousal levels will vary depending on their familiarity with background music. When consumers are more familiar with the background music they hear, they are more involved and have a stronger perception of the structural features and emotional conveyance of the music, at which point fast-paced background music can effectively increase the level of consumer arousal; When consumers are not familiar with the music they are listening to, they are less involved in the music and receive less information about the music, and thus the tempo of the background music cannot have an effective impact on their arousal. In summary, the following hypothesis is proposed:

*H3*: Consumers’ familiarity with background music moderates the effect of background music tempo on consumers’ variety-seeking behavior. Specifically, when consumers have a high level of familiarity with the background music, fast-tempo background music increases consumer arousal levels, thereby enhancing their variety-seeking behavior. However, when consumers’ familiarity with background music is low, the effect of background music tempo on consumers’ arousal and variety-seeking behavior is no longer significant.

### 2.4. Overview of studies

Five studies (including one field experiment) were conducted to test the proposed hypotheses. Study 1a and Study 1b investigated the effect and direction of background music tempo on consumers’ variety-seeking behavior by using different background music stimuli. The purpose of Study 2 is to examine the mediating role of arousal level in the influence of background music tempo on consumers’ variety-seeking behavior. Study 3 explored the moderating effect of music familiarity on the main effect. Finally, to enhance the external validity and robustness of the research, a field experiment was conducted to further validate the main effect of background music tempo on consumers’ variety-seeking behavior.

## 3. Study 1a: main effect of background music tempo on variety-seeking

### 3.1. Design and participants

One hundred and seventy-seven adults (41.55% female, mean age = 29.16) were recruited through a professional online survey platform in China (Credamo). Participants were randomly assigned to a background music condition (fast-tempo vs. slow-tempo).

### 3.2. Materials and method

#### 3.2.1. Music stimuli

We selected four groups of different types of music as background music stimuli, including Mandarin pop songs, Western pop songs, classical piano pieces, and instrumental music. Each group consisted of one fast-tempo song and one slow-tempo song. For example, in the group of classical piano pieces, we selected *Suite No. 1 In A Major, BWV 806: II. Allemande* as the fast-tempo music and *Kinderszenen, Op. 15_ No. 7 – Träumerei* as the slow-tempo music.

#### 3.2.2. Procedure

Participants were told to wear headphones and find a quiet place before starting the survey. Participants were required to listen to music throughout the completion of the questionnaire. The participants were randomly assigned to listen to one of the eight songs we selected prior.

Study 1a has two tasks. In task one, participants were asked to concentrate on listening to music for 40 s first. Then we ask participants to rate the music they were listening to in three items: “pleasantness” (“Do you think the music you are listening to now is pleasant?,” “1” = “very unpleasant” and “7” = “very pleasant”), “enjoyment” (“Do you like the music you are listening to now,” “1” = “very disliked,” “7” = “like very much”), and tempo (“What do you think of the tempo of the music you are listening to?,” “1” = “very slow,” “7” = “very fast”).

In the second task, we measured participants’ variety-seeking behavior through a consumption decision-making task. Participants were asked to imagine the following consumption scenario while listening to music: “Suppose you are going to buy a few notebooks from an online shop. You find a notebook that sells well and has positive buyer reviews. This notebook is available in two packaging options, one set includes five notebooks of the same color (color can be chosen freely) at￥9, while the other set includes five notebooks of different colors at ￥10. Which option would you prefer to buy: five notebooks of the same color or five notebooks of different colors? (“1” = “definitely buy 5 notebooks of the same color,” “7” = “definitely buy 5 notebooks of different colors”). Higher scores indicated a stronger propensity for variety-seeking. Finally, demographic information of the participants was collected.

### 3.3. Results

In this study, participants who heard 4 fast-paced songs were classified into the fast-tempo background music group, while participants who heard four slow-tempo songs were classified into the slow-paced background music group. The results showed that there were eighty-nine participants in the fast-tempo background music group and eighty-eight participants in the slow-tempo background music group.

#### 3.3.1. Manipulation check

The results of one-way ANOVA showed that participants who heard fast-tempo background music perceived the tempo of the music they listened to as faster than those who heard slow-tempo background music (*M*_fast_ = 5.30, *SD*_fast_ = 1.16; *M*_slow_ = 3.15, *SD*_slow_ = 1.56; *F* (1,175) = 109.08, *p* < 0.001, *η*^2^ = 0.38), indicating that the manipulation of background music tempo was successful.

#### 3.3.2. Main effect

Using background music tempo as the independent variable and participants’ preference for purchasing five notebooks with different colors as the dependent variable, a one-way ANOVA was conducted. The results indicated that compared to participants who heard slow-tempo background music (*M*_slow_ = 5.94, *SD*_slow_ = 1.25), participants who heard fast-tempo background music (*M*_fast_ = 6.45, *SD*_fast_ = 1.01) were more inclined to purchase notebooks with five different colors (*F* (1, 175) = 8.753, *p* = 0.004, *η*^2^ = 0.48), demonstrating a stronger tendency for variety seeking. H1 was supported.

In addition, compared to participants who heard fast-tempo background music, participants who heard slow-tempo background music generally perceived the music they heard as more pleasant (*M*_fast_ = 5.21, *M*_slow_ = 5.84, *p* = 0.001) and expressed a higher level of liking towards the music (*M*_fast_ = 5.11, *M*_slow_ = 5.90, *p* < 0.001). To exclude the potential confounding effects of these two variables, we conducted a regression analysis with background music tempo as the independent variable, consumer’s variety-seeking tendency as the dependent variable, and perceived pleasantness and liking as the control variables. The results revealed that the effect of background music tempo on consumers’ variety-seeking behavior remained significant (*p* < 0.01), while perceived pleasantness (*p* = 0.701) and liking (*p* = 0.512) of the music did not have a significant impact on consumers’ variety-seeking behavior.

### 3.4. Discussion

Study 1a examined the impact of background music tempo on consumers’ variety-seeking behavior. The results indicated that participants exposed to fast-tempo background music showed a higher tendency for variety-seeking compared to those exposed to slow-tempo background music. However, the music we selected in this study differed not only in tempo but also in various other aspects. These differences may introduce confounding to the effect of background music tempo on consumers’ variety-seeking behavior. We will address this issue in subsequent experiments.

## 4. Study 1b: main effect of background music tempo on variety-seeking

### 4.1. Design and participants

Study 1b was a one-factor (background music tempo: fast vs. slow vs. control) between-group design, where participants in the control group would not hear any background music during the experiment. One hundred and sixty-seven participants (53.29% female, mean age = 28.72) were recruited through a Chinese online survey platform (Credamo). Participants were randomly assigned to one of the three experimental groups, with 53 in the fast-tempo background music group, 56 in the slow-tempo background music group, and 58 in the no-background music group.

### 4.2. Materials and method

#### 4.2.1. Music stimuli

To minimize the differences between the two groups of music, the current experiment chose to adapt the same piece of music into two versions, fast tempo and slow tempo. Specifically, we selected a piano piece (*Silent Eyes-Fabrizio Paterlini*) with a moderate tempo through a music platform. Then, we found a professional music production company to create two versions of the music according to the musical score, one with a fast tempo of 150 BPM and another with a slow tempo of 80 BPM.

A pretest was conducted to verify whether there were differences in the perception of the tempo of the two versions of the music and to examine whether the change in tempo led to changes in the perceived pleasantness and enjoyment of the music. Forty participants (55% female, mean age = 25.45) were randomly assigned to the fast-tempo music group and the slow-tempo music group to evaluate the tempo, pleasantness, and enjoyment of the two versions of music. The results of the pretest indicated that compared to participants who listened to the slow-tempo version of the music, participants in the fast-tempo version group perceived the tempo of the music to be faster (*M*_fast_ = 5.35, *SD*_fast_ = 1.22; *M*_slow_ = 3.47, *SD*_slow_ = 0.94; *F* (1,38) = 30.130, *p* < 0.001, *η*^2^ = 0.358). However, there were no significant differences between the two music groups in terms of perceived pleasantness (*M*_fast_ = 5.67, *M*_slow_ = 5.48, *p* = 0.642) and enjoyment (*M*_fast_ = 5.19, *M*_slow_ = 4.93, *p* = 0.517). The above results indicate the appropriateness of the selected music stimuli in this experiment.

#### 4.2.2. Procedure

The experiment consists of two sessions. In the first task, participants from the two groups assigned to listen to background music were asked to concentrate on the music for 40 s, while participants in the control group were asked to meditate for 40 s. To conceal the purpose of the experiment, we told the participants that the instruction was aimed at reducing potential distractions from the external environment and enhancing their focus while completing the questionnaires. Subsequently, the two groups of participants who heard the music were required to evaluate the tempo, pleasantness, and enjoyment of the music they heard (as in Study 1a). Participants in the control group did not need to answer these questions.

The second task is a consumer decision-making task. In this session, we asked participants to imagine that they were facing the following situations while listening to music, specifically:

“You went to the supermarket today to buy some yogurt for next week. You see a certain brand of yogurt on the shelf with the following six flavors: original, peach, grape, strawberry, mango, and grapefruit. These six flavors of yogurt are priced the same. If you are going to purchase 5 boxes of yogurt for next week, please write down the quantity of each flavor of yogurt you would like to buy (You can make any combination of flavors, such as buying 5 boxes of the same flavor or buying 5 boxes of different flavors).”

We used the number of yogurt flavors finally chosen by the participants as an indicator of their variety-seeking behavior, and the more yogurt flavors participants selected, the higher their tendency towards variety-seeking. This measurement was adapted from Huang et al. (2019). In addition, we also included additional consumption decision tasks such as intertemporal choice as filler tasks to prevent subjects from guessing the true purpose of the experiment. Finally, participants’ demographic data were collected.

### 4.3. Results

#### 4.3.1. Music stimuli check

The results of the t-test revealed that participants who listened to fast-tempo background music (*M*_fast_ = 5.37, *SD*_fast_ = 0.293) perceived the tempo of the music to be faster compared to participants who listened to slow-tempo background music (*M*_slow_ = 3.52, *SD*_slow_ = 0.260, *t* (107) = 4.910, *p* < 0.01), which indicate that the manipulation of background music tempo in the experiment was successful. There were no significant differences between the two groups of participants in their evaluations of how pleasant the music was (*M*_fast_ = 5.61, *M*_slow_ = 5.48, *p* = 0.269) and how much they liked it (*M*_fast_ = 5.36, *M*_slow_ = 5.44, *p* = 0.482).

#### 4.3.2. Main effect

**Figure 1:**
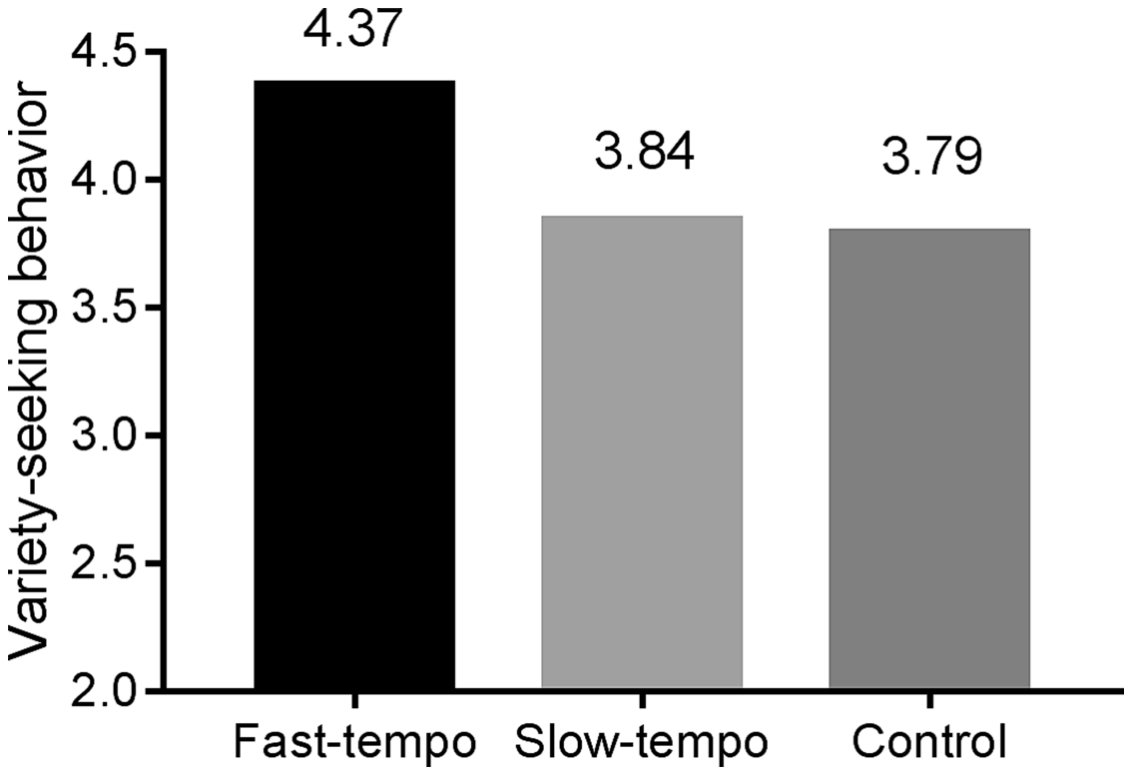
Main effect of background music tempo.

The present study employed one-way ANOVA and independent samples t-test to examine the main effects. The results (See Figure 1) showed that participants who listened to fast-tempo background music (*M*_fast_ = 4.37, *SD*_fast_ = 1.264) compared to participants who listened to slow-tempo background music (*M*_slow_ = 3.84, *SD*_slow_ = 1.430; *t* (107) = 2.046, *p* < 0.05), and those who did not listen to any background music (*M*_control_ = 3.79, *SD*_control_ = 1.475; *t* (109) = 2.214, *p* < 0.05) chose more flavors of yogurt (*F* (2,164) = 2.882, *p* < 0.05, *η*^2^ = 0.049). Participants who listened to slow-tempo background music and participants in the control group who did not listen to any background music did not show a significant difference in the number of yogurt flavors chosen (*t* (112) = 0.184, *p* > 0.1). This result indicated that participants who listened to fast-tempo background music exhibited a significantly higher tendency to variety-seeking than compared to participants who listened to slow-tempo background music and participants in the control group. Thus, H1 was supported again.

### 4.4. Discussion

Study 1b used a more rigorous manipulation of the independent variable to reduce the interference of factors other than music tempo on the experimental results. The study again confirmed the main effect of background music tempo on consumer variety-seeking behavior, supporting H1. The results show that listening to fast-tempo background music increases consumers’ variety-seeking behavior compared to listening to slow-tempo background music. Moreover, the presence of the control group suggests that the effect of background music tempo on consumers’ variety-seeking behavior is caused by fast-tempo music increasing consumers’ variety-seeking behavior rather than slow-tempo music decreasing consumers’ variety-seeking behavior.

## 5. Study 2: the mediation of arousal

### 5.1. Design and participants

Study 2 was a one-factor (background music tempo: fast vs. slow) between-group design. One hundred and thirty-six undergraduate students (57.35% female, average age = 21.24) were recruited to participate in the experiment. All participants were randomly assigned to either of the two experimental groups, with 70 in the fast-tempo music group and 66 in the slow-tempo music group.

### 5.2. Materials and method

On the day of the experiment, after participating in an unrelated consumer behavior experiment, participants were provided with the following instruction: “Next, you need to complete a music evaluation task. Please wear headphones and listen to the music throughout the entire task.”

In the first session, participants were randomly exposed to either fast-tempo or slow-tempo music (the same music stimuli as in Study 1b). All participants were asked to listen to music intently for 40 s. After that, participants were asked to continue listening to the music while completing a paper questionnaire to evaluate the pleasantness, enjoyment, and tempo of the music (same items as in Study 1a). Additionally, participants were required to report their current emotional feelings on the Affect grid, which included two dimensions: pleasure and arousal [*cf.* Russell et al. (1989)].

Next, participants were asked to complete a decision-making task measuring their variety-seeking behavior while listening to music [*cf.* Simonson (1990)]. We told the participants that in appreciation of their participation in the experiment, they would receive two gifts: paperclips and dovetail clips. They can choose any three paper clips from four shapes (star, heart, flower, diamond) and two dovetail clips from three colors (blue, green, yellow). For each gift, they are free to combine them, either choosing all the same or entirely different options. The number of shapes of paperclips chosen by the participants and the number of colors of dovetail clips chosen were summed as the indicator of their variety-seeking behavior, e.g., when a participant chose “two star-shaped paperclips, one heart-shaped paperclip, and two blue dovetail clips,” the value of variety-seeking behavior was coded as 3.

### 5.3. Results

#### 5.3.1. Music stimuli check

The results of ANOVA showed that participants who listened to fast-paced background music perceived the tempo of the music to be faster compared to participants who listened to slow-paced background music (*M*_fast_ = 5.42, *SD*_fast_ = 0.76; *M*_slow_ = 3.65, *SD*_slow_ = 1.16; *F* (1, 134) = 113.081, *p* < 0.001, *η*^2^ = 0.28), indicating that the manipulation of background music tempo was successful. There were no significant differences between the two groups of participants in their evaluations of pleasantness (*M*_fast_ = 5.86; *M*_slow_ = 5.92; *F* (1, 134) = 0.476, *p* = 0.482) and enjoyment (*M*_fast_ = 5.58; *M*_slow_ = 5.61; *F* (1, 134) = 0.614, *p* = 0.577) of the music they listened to.

#### 5.3.2. Main effect

The results of the ANOVA showed that participants who listened to fast-tempo background music chose a more total number of types of gifts compared to those who listened to slow-tempo background music (*M*_fast_ = 4.47; *M*_slow_ = 4.02; *F* (1,134) = 8.282, *p* < 0.001), indicating a significant main effect of background music tempo on consumer variety-seeking behavior. H1 was supported again.

#### 5.3.3. Mediation of arousal

**Figure 2:**
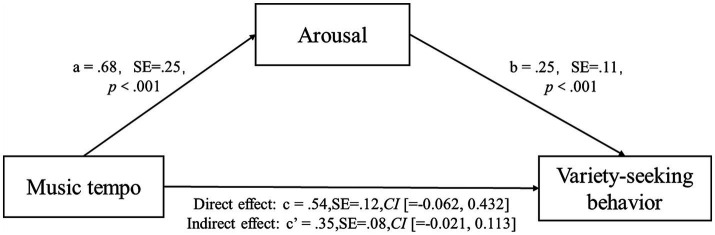
Path coefficients of the mediation analyzes in Study 2.

The results of the one-way ANOVA revealed a significant difference in arousal level between participants exposed to fast-tempo background music and those exposed to slow-tempo background music (*M*_fast_ = 5.53; *M*_slow_ = 4.75; *F* (1, 134) = 12.109, *p* < 0.001). To test the mediation role of arousal, we used PROCESS model 4 (Hayes, 2013) with 10,000 bootstrap samples and 95% confidence intervals. We included music tempo as the independent variable, arousal level as the mediator, and the indicator of variety-seeking behavior as the dependent measure. The results of the mediation analysis (See Figure 2) showed a significant indirect effect of background music tempo on consumer variety-seeking behavior through the proposed mechanism of arousal (*β* = 0.353, SE = 0.08, 95% *CI* [0.0213, 0.1132]), indicating that arousal fully mediates the effect of music tempo on consumers’ variety-seeking behavior, H2 was supported.

The results of an ANOVA revealed a significant difference in pleasure between the two groups of participants. The participants in the fast-tempo music group showed significantly higher levels of pleasure compared to those in the slow-tempo music group (*M*_fast_ = 5.46; *M*_slow_ = 5.09; *F* (1,134) = 4.063, *p* < 0.05). The mediating role of pleasure was further examined using PROCESS model 4, which showed that the indirect effect of background music tempo on consumer variety-seeking behavior through pleasure was not significant (*β* = 0.002, 95% *CI* [−0.0322, 0.0690], including 0). The result rules out the possibility of affect valence mediating the effect of background music tempo on consumers’ variety-seeking behavior.

### 5.4. Discussion

Study 2 examined the mediating role of arousal in the effect of background music tempo on consumers’ variety-seeking behavior. The results of the study indicated that listening to fast-tempo background music enhances consumer arousal, thereby increasing their variety-seeking behavior. Second, Study 2 ruled out the potential mediating effect of affect valence. Finally, Study 2 enhanced the external validity of the study by measuring participants’ real decision-making behavior in the laboratory.

## 6. Study 3: the moderation of music familiarity

### 6.1. Design and participants

Study 3 was a 2 (music familiarity: high vs. low) × 2 (music tempo fast vs. slow) between-group experimental design. We recruited 240 participants from a professional survey platform (Credamo) in China, and after excluding invalid responses, we obtained a final sample of 218 participants (57.34% female, mean age = 28.23). The participants were randomly assigned to one of four experimental groups: Fast Tempo-High Familiarity group (*n* = 54), Fast Tempo-Low Familiarity group (*n* = 54), Slow Tempo-High Familiarity group (*n* = 54), and Slow Tempo-Low Familiarity group (*n* = 56).

### 6.2. Materials and method

#### 6.2.1. Music stimuli

We chose *Kiss the Rain* and *Spring Waltz* composed by musician Yiruma as the background music stimuli for this study. These two pieces of music share a similar style, but *Kiss the Rain* is widely known, while *Spring Waltz* is relatively obscure. Similarly, following the procedure in Experiment 1b, we commissioned a professional music production company to create two versions (fast-paced and slow-paced) based on the score of these two songs. The fast-tempo version of the two songs had a tempo of about 150 BPM, while the slow-tempo version of the two songs had a tempo of about 80 BPM.

Similar to Study 1b, we conducted a pretest (122 participants, 47.54% female, mean age = 25.72). Pretest results indicated that participants were significantly more familiar with *Kiss the Rain* than with *Spring Waltz* (*M*_kiss_ = 6.04, *SD*_kiss_ = 2.31; *M*_spring_ = 3.72, *SD*_spring_ = 0.99; *F* (1,120) = 51.257, *p*<0.001, *η*^2^ = 0.032). There were no significant differences in the conveyed emotions (*p* > 0.05), pleasantness (*p* > 0.05), and enjoyment (*p* > 0.05) between the two music pieces.

Furthermore, the results also indicated that the two versions of *Kiss the Rain* with different tempos only differed significantly in terms of tempo (*M*_fast_ = 5.57, *SD*_fast_ = 2.72; *M*_slow_ = 3.44, *SD*_slow_ = 2.14; *F* (1,60) = 11.792, *p* < 0.001, *η*^2^ = 0.038). There were no significant differences observed in terms of conveyed emotions (*p* > 0.1), pleasantness (*p* > 0.05), enjoyment (*p* > 0.05), and familiarity (*p* > 0.05). Similarly, the two versions of *Spring Waltz* with different tempos also showed significant differences only in terms of tempo ratings (*M*_fast_ = 5.49, *SD*_fast_ = 1.66; *M*_slow_ = 3.47, *SD*_slow_ = 1.94; *F* (1,58) = 19.046, *p* < 0.001, η^2^ = 0.053), while no significant differences were found in other items (all *p* > 0.05). These findings indicate that the selected music stimuli in this experiment are reasonable and can be used for the subsequent study.

#### 6.2.2. Procedure

The procedure of Study 3 was similar to that of Study 1b. The difference is that we added a measurement of participants’ familiarity with the music, which was measured using a single item: “Are you familiar with the music you are listening to? (7-point scale: “1” = “Not familiar at all,” “7” = “very familiar”).” Additionally, we measured participants’ affective valence and arousal level. This included two items: “How pleasant do you feel right now?” (9-point scale: “1” = “Extremely unpleasant,” “9” = “Extremely pleasant”) and “How excited do you feel right now?” (9-point scale: “1” = “Extremely unexcited” “9” = “Extremely excited”) [*cf.* Russell et al. (1989), Huang et al. (2019)].

The measurement of participants’ variety-seeking behavior in this experiment was similar to that of Study 1b. However, the yogurt used in Study 1b was replaced with a real product—*Pocky*. We presented participants with a picture containing seven boxes of *Pocky* with different flavors to enhance the realism of the decision-making scenario. In the experiment, participants were asked to choose any five boxes of *Pocky* from a set of 7 flavors presented in the picture. The number of flavors ultimately selected by participants was used as the indicator of their variety-seeking behavior.

### 6.3. Results

#### 6.3.1. Music stimuli check

The results of ANOVA showed that participants were significantly more familiar with *Kiss the Rain* than with *Spring Waltz* (*M*_kiss_ = 5.86; *M*_spring_ = 3.42; *F* (1,216) = 257.442, *p* < 0.001). Participants who listened to the fast-tempo version of *Kiss the Rain* perceived the tempo of the music to be faster compared to those who listened to the slow-tempo version (*M*_fast_ = 5.53; *M*_slow_ = 3.11; *F* (1,106) = 135.283, *p* < 0.001). Similarly, participants who listened to the fast-tempo version of *Spring Waltz* perceived the tempo of the music to be faster compared to those who listened to the slow-tempo version (*M*_fast_ = 5.47; *M*_slow_ = 3.09; *F* (1,108) = 142.448, *p* < 0.001). There were no significant differences in the pleasantness and enjoyment between the two versions of *Kiss the Rain* (*p* > 0.1). The same applies to *Spring Waltz*. The results indicated that the music stimuli choose for music familiarity and music tempo was suitable and could be used for the study to be conducted next.

#### 6.3.2. Moderation of music familiarity

**Figure 3:**
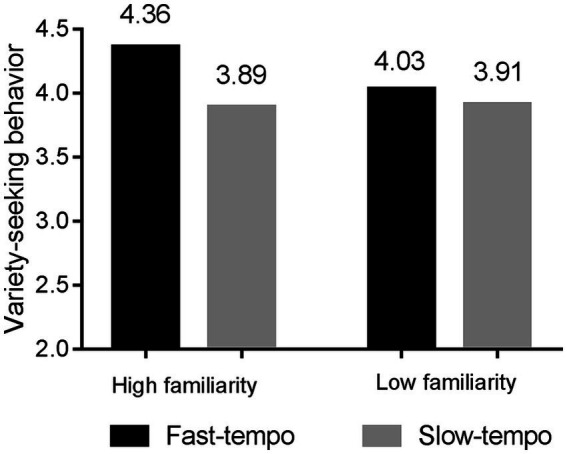
Moderating role of music familiarity.

The results of the ANOVA analysis indicated a significant interaction effect between background music tempo and music familiarity on consumers’ variety-seeking behavior (*F* (3, 214) = 6.374, *p* = 0.017, *η*^2^ = 0.07) (see Figure 3). When exposed to familiar music, participants who listened to the fast tempo version exhibited a significantly higher tendency for variety-seeking (*M*_fast_ = 4.36, *SD*_fast_ = 0.89) compared to those who listened to the slow tempo version (*M*_slow_ = 3.89, *SD*_slow_ = 0.84; *F* (1, 106) = 7.644, *p* = 0.007, η^2^ = 0.02). When exposed to unfamiliar music, there was no significant difference in variety-seeking behavior between the two groups (*M*_fast_ = 4.03, *SD*_fast_ = 1.15; *M*_slow_ = 3.91, *SD*_slow_ = 1.15; *F* (1, 108) = 2.38, *p* = 0.672).

**Figure 4:**
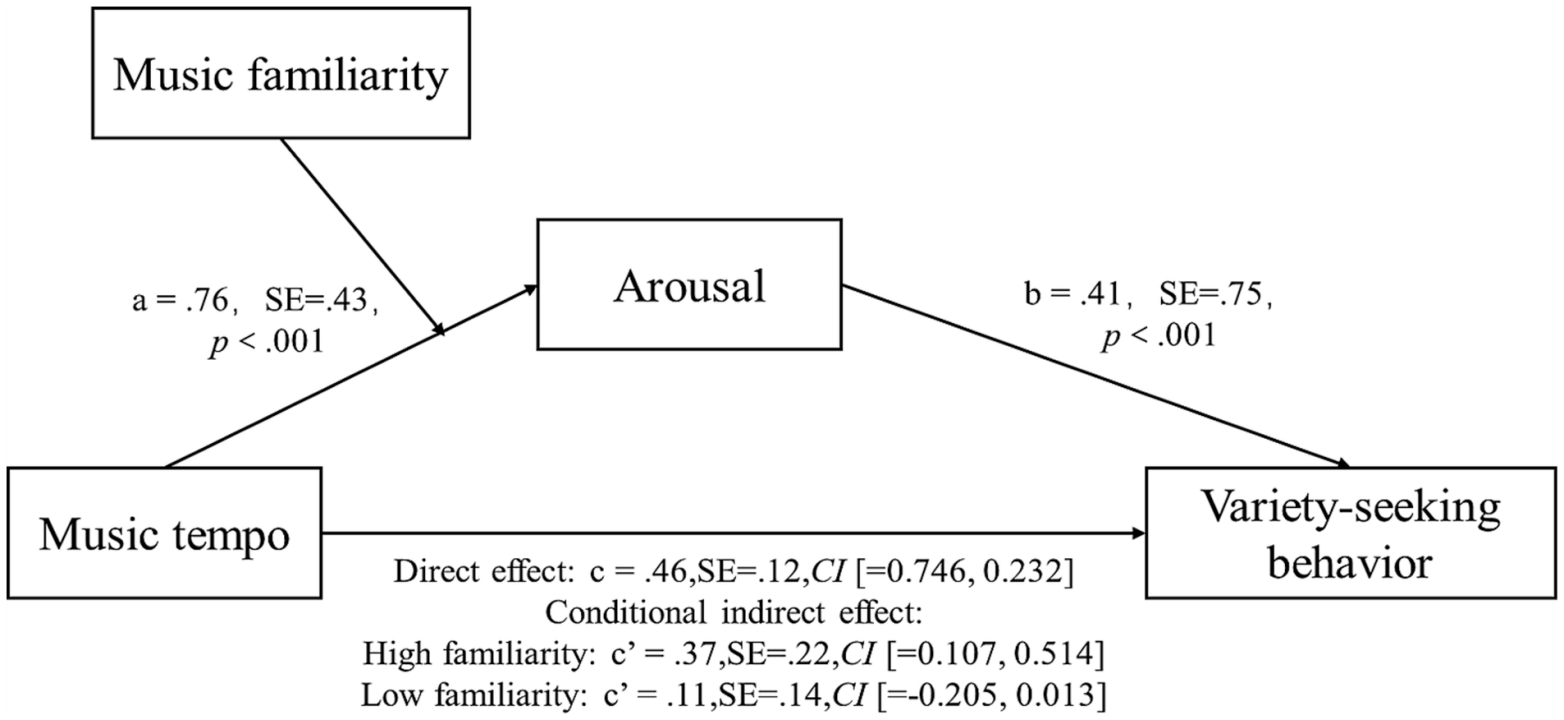
Path coefficients of the moderated mediation analyzes in Study 3.

To test the moderating effect of music familiarity, we conducted a moderated mediation analysis [model 7 in PROCESS with 5,000 Bootstrap samples and 95% confidence intervals, Hayes (2013)]. The results (see Figure 4) revealed a significant overall mediation effect of arousal (*β* = 0.46, SE = 0.12, 95% *CI* [0.7462, 0.2323]). Specifically, the indirect effect of background music tempo on consumers’ variety-seeking behavior through arousal level was significant in the high music familiarity condition (*β* = 0.37, SE = 0.22, 95% *CI* [0.1071, 0.5140]). However, this indirect effect was not significant in the low music familiarity condition (*β* = 0.11, SE = 0.14, 95% *CI* [−0.2053, 0.0131]). Thus, music familiarity moderated the influence of music tempo on variety-seeking behavior, H3 was supported.

An ANOVA analysis showed that the background music tempo did not have a significant effect on the participants’ affect valence, regardless of their familiarity with the music they were listening to. We employed Model 7 in the PROCESS to examine further and found that the mediating effect of affect valence was not significant. The interval of the mediating effect of affect valence contained 0 regardless of participants’ familiarity with the music.

### 6.4. Discussion

Study 3 manipulated participants’ familiarity with the background music by using music with different levels of popularity and examined the moderating role of music familiarity in the impact of background music tempo on consumers’ variety-seeking behavior (H3). Specifically, when consumers’ familiarity with the music they listen to is high, the tempo of the background music has a significant impact on their variety-seeking behavior. However, when consumers’ familiarity with the music they listen to is low, the effect of background music tempo on consumers’ variety-seeking behavior attenuated. In addition, Study 3 enhanced the external validity of the main effect by using a real food brand to measure consumer variety-seeking behavior.

## 7. Study 4: a field study

### 7.1. Design and participants

We conducted the field experiment in an ice cream shop in China. The main product of this store is Yogurt ice cream, and three new flavors of Yogurt ice cream are introduced on the first day of each month. We chose to conduct this study during the peak season of the store, specifically from June 1st, 2022, to August 31st, 2022, spanning a total of 92 days.

### 7.2. Materials and method

#### 7.2.1. Music stimuli

We collected 120 popular piano songs from a music platform as background music stimuli for this experiment, including thirty classical piano masterpieces (e.g., *Wedding in a Dream-Richard Clayderman, Für Elise – Beethoven*) and piano accompaniment for ninety Chinese and foreign pop songs (e.g., *Blue and White Porcelain – Jay Chou*, *Encounter – Stephanie Sun, Suddenly Missing You – Mayday*). We manipulated the tempo of these songs by altering their playback speed， with the fast tempo group playing these songs at 1.8x speed and the slow tempo group playing these songs at 0.7x speed.

#### 7.2.2. Measurement of consumer variety-seeking behavior

Consumers’ purchase of unfamiliar products or brands as a switching behavior is also a manifestation of variety-seeking behavior (Kwon et al., 2023). In this study, customers’ purchase of new flavor yogurt ice cream was seen as diversified purchase behavior. We use the ratio of the number of units sold of the new flavor of yogurt ice cream to the total number of units sold of yogurt ice cream on that day as the indicator of consumers’ variety-seeking seeking behavior.

#### 7.2.3. Procedure

Starting from June 1st, 2022, we instructed the store staff to randomly play the selected 120 songs through the in-store audio system during the daily business hours (9:00 a.m. – 10:00 p.m.), alternating at 1.8x and 0.7x speed each day. We categorized the days when music was played at 1.8x speed as the fast-tempo group and the days when music was played at 0.7x speed as the slow-tempo group. Both groups consisted of 46 days.

Additionally, we recorded the number of new-flavor yogurt ice cream sold at the store each day, as well as the total number of yogurt ice cream sold on that day. Then, we calculated the ratio of new-flavor yogurt ice cream to the total number of yogurt ice cream sold on that day as a measure of consumers’ variety-seeking behavior.

### 7.3. Results

Using the proportion of new-flavor yogurt ice cream servings to the total servings of yogurt ice cream sold by the store each day as the dependent variable, and the tempo of music played in the store each day as the independent variable, an ANOVA was conducted. The results showed that when the music was played at a fast tempo in the store, the proportion of new-flavor yogurt ice cream servings to the total servings of yogurt ice cream sold each day was significantly higher compared to when the music was played at a slow tempo (*M*_fast_ = 0.649, *SD*_fast_ = 0.17; *M*_slow_ = 0.527, *SD*_slow_ = 0.18; *F* (1, 90) = 11.164, *p* = 0.001). This result indicated that the fast-tempo background music will make customers more willing to buy new flavors of yogurt ice cream, that is, to make more diverse choices. H1 was supported again.

### 7.4. Discussion

Study 4 observed consumers’ variety-seeking behavior in a real consumption scenario and replicated the main effect of background music tempo on consumer variety-seeking behavior, which enhanced the generalizability and external validity of the research.

## 8. General discussion

### 8.1. Conclusion

The present research systematically explores the effect of background music tempo on consumers’ variety-seeking behavior and its underlying mechanisms. Through five experiments, we tested the three hypotheses we proposed. Study 1a, 1b, and 4 (a field study) explored the causal relationship between background music tempo and consumer variety-seeking using different background music stimuli and measurements of consumer variety-seeking behavior and showed that fast-tempo background music increases consumer variety-seeking behavior. Study 2 examined the mediating role of arousal in the effect of background music tempo on consumers’ variety-seeking behavior and ruled out alternative explanations of affect valence. The results demonstrated that fast-tempo background music increases consumers’ variety-seeking behavior by enhancing their arousal. Study 3 manipulated participants’ familiarity with background music by using music with different levels of popularity, verifying the moderating effect of music familiarity. We found that the tempo of background music affects consumers’ arousal and subsequent variety-seeking behavior only when consumers are familiar with the background music they listen to. When consumers are unfamiliar with the background music, the effect of background music tempo on consumers’ variety-seeking behavior is weakened.

### 8.2. Theoretical contributions

Our research provides several theoretical contributions. First, our findings shed light on sensory marketing, especially auditory marketing. Music plays a vital role in auditory marketing. Previous research mainly concentrated on how to evoke consumers’ hedonic feelings by using music in advertisements and consumer environment to enhance consumers’ positive attitude to brand [see a review, Meyers-Levy et al. (2011)]. In addition, some of the existing research concern on consumers’ most primal sensorial response to music (Roballey et al., 1985; Brodsky, 2002; Su et al., 2023). Limited attention was paid to the influence of music on consumer specific decision-making behavior. Our research examined the impact of music tempo on consumers’ variety-seeking behavior (one of the most important aspects of consumer behavior) and its underlying mechanism, finding that fast-tempo music increases consumers’ variety-seeking behavior by increasing consumers’ arousal levels. By shedding light on this novel relationship, our research provides new insights to the field of auditory marketing.

Second, our research expanded the range of environmental factors that influence consumers’ variety-seeking behavior. In a review, Zhang (2022) points out that most past research concentrated on internal factors influencing consumption variety-seeking, thus, additional research is needed to widely and deeply explore the external factors. The current research examined the impact of background music (an important and common element in the shopping environment) tempo on consumers’ variety-seeking behavior, enriching the existing literature on the influence of external factors.

Third, this research enhanced understanding of arousal as the underlying mechanism. In previous research on the effects of music on consumer emotion and behavior, most articles focused on the mediating role of emotional valence (Areni, 2003; Michel et al., 2017), with limited consideration of arousal. This study revealed that arousal is the underlying mechanism through the effect of background music tempo on consumers’ variety-seeking behavior rather than affect valence, enriched the literature on arousal. On the other hand, there are inconsistencies in the existing literature about the personal arousal level as the underlying mechanism of variety-seeking behavior. Roehm and Roehm (2005) found that people seek more variety at low arousal than high arousal moments. In contrast, Gullo et al. (2019) pointed out that individuals’ variety-seeking is lower in the early morning due to the lower arousal. In our research, the results showed that the high arousal state triggered by fast-tempo music increased consumers’ variety-seeking behavior, which supports the conclusion that arousal positively affects variety-seeking.

Forth, our research extended the understanding of music familiarity. Familiarity exerts a very powerful force over music perception. Existing research has primarily focused on the direct impact on individuals (Pereira et al., 2011; Hee Park et al., 2014), with a limited examination of the interaction between music familiarity and other music structural characteristics. This research revealed the moderating role of music familiarity in the effect of background music tempo on consumers’ variety-seeking behavior, providing new insights into the study of music familiarity.

### 8.3. Management implications

The findings of this research provide important managerial implications for marketers. First, the results have implications for in-store background music design. For example, salespeople in supermarkets always need to promote products with a variety of choices (e.g., yogurt with different flavors, shampoos with different fragrances), and they could choose to play fast-tempo music that consumers are more familiar with to increase consumers’ purchase intentions. Additionally, for shops with frequent product updates (e.g., Zara, UNIQLO), it may be wise for managers to play fast-tempo background music in the store on new product launch days to encourage customers to buy more new offerings.

Second, our findings could help marketers choose appropriate music for product ads. For example, in the online shopping scenario, when an e-commerce company needs to promote or sell new products, the marketers can use fast-tempo background music on the product display page to increase consumers’ purchase intention of products they had never bought. At the same time, according to the consumer’s membership information (age, gender, education level, race, etc.), the background music can be customized when consumer browses the product page. For example, playing fast-tempo music for different generations that they are familiar with, playing the songs of Beatles for the 70s and 80s, and Korean hip-pop for the 00s.

Third, our findings provides new insights for personalized recommendation advertising. For instance, many online video sites and music sites now target their audiences with ads, and if an audience is detected listening to fast-tempo music, the platform can recommend more ads of novel products and products with variety choices to him/her.

### 8.4. Limitations and future research

The present research also has certain limitations that warrant further exploration in future research. First, the current research only roughly divided the tempo of background music into two dimensions: fast and slow. However, the tempo of a song is not consistently fast or slow but has certain variations. Future research could explore the effect of fluctuations in music tempo on consumer psychology and behavior by using more different kinds of music. Moreover, the emotions and meanings conveyed by music are influenced by all structural features. Future research could also explore the interactive effects of music tempo with other music structural features on consumer psychology and behavior, such as the interaction between music tempo and pitch, and the interaction between music tempo and timbre.

Second, the present study only focused on the separate influence of music tempo, but the real shopping environments are more complex. In the future, researchers could conduct more field experiments in different shopping scenarios to explore the interaction effects of music tempo with other auditory and visual elements in the environment. For example, in online shopping scenarios, future research could explore the interaction effect between background music tempo and video narration speech rate of online ads on consumer variety-seeking behavior. In offline scenarios, more research could explore how the tempo of background music interacts with the color tone of the environment, the brightness of the light, and the speed of salespeople’s speech.

Third, more boundary conditions could be explored in the future. For example, the product category may be a moderating variable in this research. Specifically, when consumers purchase high-involvement products (e.g., cars, computers), they engage in detailed information processing (Vaughn, 1980) and are more likely to adopt the central persuasion path. In such cases, environmental cues like background music tempo hardly have an impact on consumers’ decision-making behavior. On the other hand, for low-involvement products (e.g., yogurt, biscuits), consumers rely more on emotions and peripheral cues and in these instances, background music tempo will significantly influence consumers’ decision-making behavior.

Finally, all subjects in the current research were recruited from China, future research could replicate our experiments in different countries and different cultures to explore the generalization of the impact of music tempo on variety-seeking behavior. For example, for Latinos who are accustomed to listening to fast-tempo music, will fast-tempo music also increase their variety-seeking behavior?

## Data availability statement

The raw data supporting the conclusions of this article will be made available by the authors, without undue reservation.

## Ethics statement

Ethical review and approval were not required for the study on human participants in accordance with the local legislation and institutional requirements. The participants provided their written informed consent to participate in this study.

## Author contributions

All authors listed have made a substantial, direct, and intellectual contribution to the work and approved it for publication.

## Conflict of interest

The authors declare that the research was conducted in the absence of any commercial or financial relationships that could be construed as a potential conflict of interest.

## Publisher’s note

All claims expressed in this article are solely those of the authors and do not necessarily represent those of their affiliated organizations, or those of the publisher, the editors and the reviewers. Any product that may be evaluated in this article, or claim that may be made by its manufacturer, is not guaranteed or endorsed by the publisher.
